# Thoracic CT Screening in a Population With Unidentified Lung Cancer Risk Factors: Does It Facilitate the Early Diagnosis of Lung Cancer?

**DOI:** 10.1155/carj/7611259

**Published:** 2025-10-29

**Authors:** Aysegul Gencer, Buket Caliskaner Ozturk, Gizem Senkardesler, Ersan Atahan, Bilun Gemicioglu, Sermin Borekci

**Affiliations:** ^1^Department of Respiratory Medicine, Cerrahpasa Faculty of Medicine, Istanbul University-Cerrahpasa, Istanbul, Turkey; ^2^Department of Respiratory Medicine, Kırklareli Education and Research Hospital, Kırklareli, Turkey

**Keywords:** early-stage lung cancer, lung cancer risk factors, lung cancer screening, pulmonary nodules, thorax CT screening

## Abstract

**Background:**

Lung cancer is the predominant cause of cancer-related mortality globally. Computed tomography (CT) scanning is employed to enhance the early diagnosis of lung cancer by screening for risk factors. This study aimed to examine the impact of CT scanning on the incidence of lung cancer detection in a group with unidentified risk factors for the disease.

**Methods:**

Data from two patient cohorts were analyzed: the “study group,” comprising individuals with a CT-detected pulmonary nodule of ≥ 8 mm and unknown lung cancer risk factors, and the “control group,” consisting of individuals with a pulmonary nodule of ≥ 8 mm identified by CT scan due to known lung cancer risk factors.

**Results:**

No significant difference was seen between the groups regarding malignancy frequency (*p*=0.155) and early-stage occurrence (*p*=0.842).

**Conclusions:**

The incidence of lung cancer in pulmonary nodules measuring ≥ 8 mm is not influenced by the presence of lung cancer risk factors.

## 1. Introduction

In 2020, global data indicated that 265.6 million individuals across all age groups were diagnosed with cancer, resulting in approximately 182.8 million cancer-related untimely deaths. Among these malignancies, 124.3 million are avoidable, and 58.5 million are treatable [1]. Lung cancer is the largest cause of cancer-related mortality, responsible for 1.8 million deaths [2, 3]. The mortality rate is predicated on an advanced-stage diagnosis.

The utilization of computed tomography (CT) has markedly enhanced the detection of pulmonary nodules, proving beneficial for the early diagnosis of lung cancer and the decrease of mortality rates. The US National Lung Screening Trial (NLST) demonstrated that lung cancer screening via CT can decrease lung cancer mortality by 20% [4, 5]. Recent data confirm that the frequency of early-stage lung cancer detection has increased following the introduction of CT [6].

Studies on lung cancer screening have been performed for the early detection of lung cancer using CT. In these screening models, risk variables for lung cancer have been delineated to optimally select the population for early detection screening [7]. Identifying these risk variables delineates the population eligible for screening and mitigates the risks associated with overdiagnosis, intrusive interventions, and radiation exposure [8].

Numerous published studies exist regarding lung cancer risk factors [4, 9–11]. Studies also detail the incidence of lung cancer in patients who got CT scans for unrelated reasons, irrespective of lung cancer risk factors [12]. Nevertheless, a substantial population has not undergone CT screening within a defined timeframe, such as during the coronavirus pandemic period. No research exists regarding the incidence of lung cancer in pulmonary nodules identified during the period when a significant portion of the population underwent CT screening at a specified time.

Thorax CT examinations were commonly utilized in the management of patients throughout the COVID-19 pandemic. The utilization of thorax CT in emergency departments rose by 88.6% from the prepandemic period to the COVID-19 pandemic. CT examinations in younger and female patients have significantly increased compared to the prepandemic period [13, 14].

Studies have found a higher percentage of early-stage lung cancer cases among patients diagnosed through large-scale chest CT scans performed during the COVID-19 pandemic [15].

This study aimed to examine the prevalence of lung cancer in pulmonary nodules measuring ≥ 8 mm on thoracic CT among patients with unidentified lung cancer risk factors, as well as to assess its impact on the rate of early-stage lung cancer detection.

## 2. Methods

### 2.1. Study Design and Setting

The study protocol was established as a retrospective analysis. Two patient cohorts were designated. From March 11, 2020, to March 11, 2022, a “study group” was established including patients with unidentified lung cancer risk factors who received thorax CT scanning due to the COVID-19 pandemic and were incidentally found to have pulmonary nodules measuring ≥ 8 mm. From March 11, 2018, to March 10, 2020, a “control group” was intended to be established from patients who received thorax CT scans at the chest illnesses outpatient clinic due to lung cancer risk factors, with pulmonary nodules measuring ≥ 8 mm identified. The study group consisted of patients who underwent chest CT scans during the pandemic to exclude COVID-19, whose lung cancer risk factors (history of smoking, previous cancer diagnoses, familial cancer history, genetic polymorphisms, exposure to asbestos, exposure to ionizing radiation, occupational hazards, prolonged exposure to severe air pollution, and chronic respiratory diseases) were unknown and had not been investigated, and whose potential hidden exposure status was unknown (e.g., passive smoking, environmental, and occupational).

This study was approved by the local clinical research ethics committee (no: E-83045809-604.01.01-381251). Written informed consent was obtained from all included patients.

### 2.2. Participants

#### 2.2.1. Control Group

##### 2.2.1.1. Inclusion Criteria

- From March 11, 2018, to March 10, 2020, a pulmonary nodule measuring ≥ 8 mm was identified on a thoracic CT scan conducted due to the existence of lung cancer risk factors.
- The risk factors for lung cancer include advanced age (50–90 years), male sex, history of smoking, previous cancer diagnoses, familial cancer history, genetic polymorphisms, exposure to asbestos, exposure to ionizing radiation, occupational hazards, prolonged exposure to severe air pollution, and chronic respiratory diseases [7, 16, 17].
- Regular follow-up visits for a minimum of 2 years for the monitoring of lung nodules subsequent to an initial CT scan.
- To have given written consent to the volunteer consent form.

##### 2.2.1.2. Exclusion Criteria

- History of lung cancer or any malignancy.
- Presence of active lung infection.
- Diagnosis or suspicion of interstitial lung disease.
- Pulmonary vascular disease.
- Pleural diseases and pleural effusion.
- Pregnancy.
- Chronic diseases such as heart failure and kidney failure.

#### 2.2.2. Study Group

##### 2.2.2.1. Inclusion Criteria

- From March 11, 2020, to March 10, 2022, a lung nodule measuring ≥ 8 mm was identified on a thoracic CT scan conducted at our institution during the COVID-19 pandemic.
- Regular follow-up visits for a minimum of 2 years for the monitoring of lung nodules subsequent to the initial CT scan.
- To have given written consent to the volunteer consent form.

##### 2.2.2.2. Exclusion Criteria

- Diagnosed with COVID-19 at the time of thorax CT scan.
- Parenchymal findings suggestive of COVID-19 at the time of thorax CT scan.
- Positive SARS-CoV-2 PCR test performed simultaneously.
- History of lung cancer or any malignancy.
- Presence of active lung infection.
- Diagnosis or suspicion of interstitial lung disease.
- Pulmonary vascular disease.
- Pleural diseases and pleural effusion.
- Pregnancy.
- Chronic diseases such as heart failure and kidney failure.

### 2.3. Variables and Data Collection

Demographic and anthropometric data were obtained from patients' files.

### 2.4. Thorax CT Scans

Patients diagnosed with COVID-19 or patients with parenchymal findings supporting COVID-19 were not included in the study group. Both groups exhibited noncalcified solid pulmonary nodules in the lung parenchyma on baseline CT scans. The study contained data from a single nodule per patient. In individuals with multiple pulmonary nodules, data from the largest pure solid lung nodule were utilized. Data pertaining to lung nodules exhibiting a calcification pattern, subsolid characteristics, and ground-glass components were removed. Data from patients undergoing routine CT follow-up at intervals based on the greatest diameter of the long axis of the biggest nodule were included. Any nodule deemed benign through consistent monitoring was included in the data if it exhibited no changes for a minimum of 2 years.

Pulmonary nodules on thorax CT were divided into 4 groups according to their size: 8–10, 11–20, 21–30, and > 30 mm.

### 2.5. Diagnosis of Malignancy

Patients diagnosed with lung cancer through histological assessment of biopsy specimens were categorized according to the World Health Organization categorization of lung neoplasms [18].

### 2.6. Staging

The positron emission tomography–CT (PET-CT) results and staging information for patients diagnosed with malignancy were categorized into early stage, locally advanced stage, and advanced stage, following the European Society for Medical Oncology (ESMO) guidelines [19, 20].

### 2.7. Study Sample

All patients who fulfilled the criteria and provided consent were enrolled in the study.

### 2.8. The Power Analysis

In the planned study, an effect size of 0.30 is anticipated, with an alpha significance level of 0.05% and 95% power. The sample size was calculated to be 301 patients in total, with 158 patients in the “control group” and 143 patients in the “study group.”

### 2.9. Statistical Analysis

The analysis was conducted using IBM's Statistical Package for the Social Sciences (SPSS) Version 22.0. Categorical data were presented as frequency and percentage, and numerical variables were summarized using mean ± standard deviation. The Mann–Whitney U test was employed for pairwise comparisons of the groups with non-normally distributed variables. Chi-square and Fisher's exact tests were employed to assess categorical variables.

## 3. Results

This study comprised 301 patients, with 52.5% (*n* = 158) assigned to the control group (patients with risk factors) and 47.5% (*n* = 143) assigned to the study group (patients with unknown risk factors). The control group exhibited a significantly higher mean age compared to the study group (*p*=0.009). No significant difference was observed between the two groups when comparing the gender variable (*p*=0.422) (Table 1).

**Figure 1:**
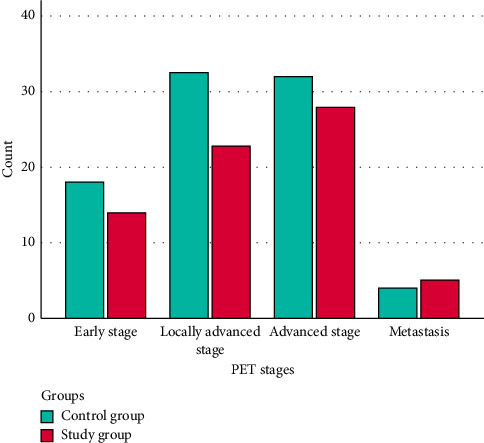
Comparison of groups by PET stages.

A significant difference was observed between the groups regarding pulmonary nodule sizes (*p*=0.037). In the control group, 68.3% of patients had pulmonary nodules sized between 21 and 30 mm, while 57.1% of patients in the study group had nodules sized between 8 and 10 mm. No significant differences were observed between the groups regarding malignancy frequency (*p*=0.155), staging (*p*=0.842), and histopathologic diagnosis (*p*=0.289) (Table 2) (Figure 1).

**Figure 2:**
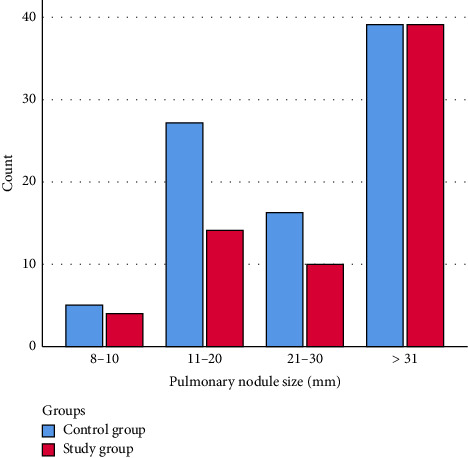
Comparison of patients with malignancy by pulmonary nodule sizes.

**Figure 3:**
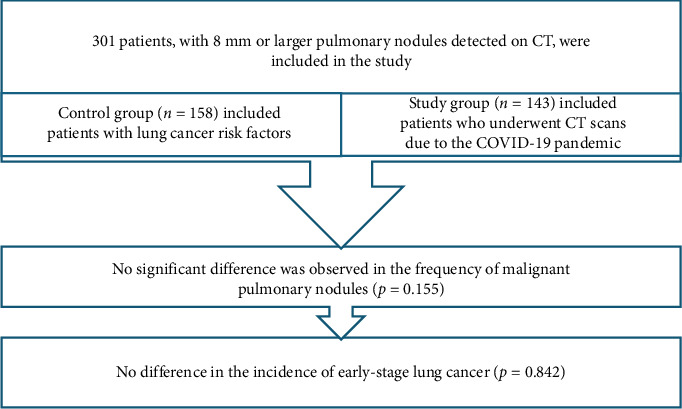
Flowchart of the study.

A significant difference was observed between malignancy frequency and pulmonary nodule size (*p*=0.001). Notably, 85.7% of patients with nodules sized 8–10 mm and 63.1% of those with nodules sized 11–20 mm did not exhibit malignancy. Malignancy was observed in 63.4% of patients with pulmonary nodules measuring 21–30 mm and in 90.7% of patients with nodules exceeding 31 mm. No significant difference in malignancy frequency was observed between the groups when comparing pulmonary nodule size (*p* > 0.05) (Table 3) (Figures 2 and 3).

Among the cohort of patients with risk factors monitored for a minimum of 2 years, 87 were diagnosed with malignancy. Of these, 51 were identified postinitial CT scan, whereas 36 were diagnosed based on nodule size during subsequent follow-up evaluations. In the cohort with unidentified risk factors, 70 patients received a malignancy diagnosis: 48 were identified postinitial CT, while 22 were diagnosed based on nodule size during follow-up assessments.

## 4. Discussion

This study examined the incidence of lung cancer and early-stage lung cancer in pulmonary nodules measuring ≥ 8 mm between a cohort of individuals with established lung cancer risk factors and a population with unidentified lung cancer risk factors. Nonetheless, no substantial difference was observed between the two groups for the incidence of lung cancer or early-stage lung cancer. The average age of the group with unidentified risk factors was lower, and lung nodules measuring 8–10 mm were prevalent in this cohort. Nodules measuring 21–30 mm were more prevalent in the group with risk factors. Nonetheless, there was no variation in the incidence of lung cancer diagnoses and their stages.

The Evidence-Based Clinical Practice Guidelines of the American College of Chest Physicians advocate for the evaluation of malignancy risk in pulmonary nodules. The therapeutic strategy for 8–10 mm pulmonary nodules outlines the methodology for evaluating the clinical likelihood of cancer. The factors employed to evaluate the likelihood of malignancy encompass age, smoking history, cancer background, nodule dimensions, irregular margins, upper lobe positioning, growth progression, and FDG-PET scan activity. The Fleischner Society 2017 guideline cites this work about the management of solid lung nodules measuring ≥ 8 mm [21, 22]. Our study assessed the incidence of lung cancer in patients with pulmonary nodules of ≥ 8 mm, disregarding the presence or absence of the recommended criteria, and determined that the results were indistinguishable from the incidence of lung cancer in patients meeting the criteria. This finding suggests that the occurrence of malignancy is constant in lung nodules measuring ≥ 8 mm, irrespective of the presence of risk factors. When addressing these nodules, it may be advantageous to simplify the method rather than employing complex algorithms or extensive criteria for malignancy likelihood. Additional research in this area is required.

In our study, patients diagnosed with COVID-19 or those with findings suggestive of COVID-19 on CT (e.g., ground-glass opacity) were excluded, and solid nodules of 8 mm or larger were included. In a study by He et al. that included pulmonary nodules in patients during the COVID-19 pandemic, although screening during the COVID-19 outbreak led to an increase in the detection rate of solid nodules in practice, no increase was observed in high-risk nodules (potentially malignant nodules), which supports the findings of our study [23].

We assessed the prevalence of malignancy in lung nodules measuring ≥ 8 mm in a population with unidentified risk factors and determined it to be elevated in our study. The 2017 guidelines of the Fleischner Society also endorse this. Advanced imaging techniques, including CT, PET-CT, or tissue biopsy, are advised 3 months following the identification of a solitary solid lung nodule measuring ≥ 8 mm, irrespective of risk classification (low or high). A threshold value of 6 mm is established for numerous nodules, necessitating follow-up for those measuring 6 mm or above. Nonetheless, when a bigger nodule is present, therapy should adhere to the standards applicable to single nodules. The phrase “larger nodule” is not delineated in this section of the guideline [22]. Based on our study, pulmonary nodules of ≥ 8 mm can be considered at this point. Furthermore, a simplified approach for nodules ≥ 8 mm will alleviate clinicians' uncertainties, preventing overdiagnosis and unnecessary interventions.

The 2017 recommendations of the Fleischner Society reference the NELSON study in their examination of numerous nodules. The guidelines indicate that the risk of primary cancer escalates with an increase in the total number of nodules from 1 to 4. The NELSON trial indicated that an increase in the number of nodules correlated with a statistically insignificant trend towards a heightened likelihood of lung cancer, suggesting that the risk of malignancy did not significantly differ based on the number of nodules, and that the initial nodule count could not effectively differentiate between benign and malignant nodules [9]. Furthermore, the NELSON study did not evaluate nodule size in correlation with nodule count. This study will yield significant insights for subsequent research in this domain.

McWilliams et al. observed a malignancy frequency of 5.5% in pulmonary nodules, encompassing all nodules detectable on CT, including those measuring ≥ 8 mm and those smaller than 8 mm. The malignancy rate in lung nodules measuring < 8 mm was not analyzed. Our study examined the prevalence of lung cancer in pulmonary nodules of ≥ 8 mm, revealing an incidence range of 43.5%–56.5% [10]. Literature data supporting these rates are also available [24–26].

McWilliams et al. assessed a total of 144 malignant nodular cases. The mean size of malignant nodules was established to be 13.9–15.7. The results indicated a nonlinear connection between nodule size and lung cancer [10]. We assessed a total of 157 malignant nodular cases and examined them based on their dimensions. A notable rise in malignancy was detected in nodules over 20 mm, aligning with the previously cited results. This study also observed a rise in lung cancer incidence with advancing age. Despite the reduced age in the cohort with unidentified risk factors, it remained above 60 years.

Li et al. conducted a study in China from 2015 to 2021 that assessed the incidence of lung cancer among patients detected using low-dose CT. The rates were shown to be similar in those at elevated risk for lung cancer and those lacking identifiable high-risk factors. They underscored the imperative for prospective studies to assess the efficacy of CT screening and the significance of identifying high-risk characteristics or prescreening biomarkers in individuals deemed not high-risk [12]. This study supports our results that the incidence of lung cancer is elevated in a cohort of patients with unidentified risk factors. Our study indicates that assessing risk factors for lung cancer in patients with pulmonary nodules measuring ≥ 8 mm is unnecessary.

As stated in the NLST trial, the increase in CT use has led to an increase in the detection of early-stage lung cancer. Wang et al. analyzed data from 72 lung cancer patients who were randomly identified during the COVID-19 pandemic and found that 36.1% of them were in the early stages. It was also reported that 63.8% of the patients were classified as being in the advanced stages. However, they did not include a control group to determine the rate of early-stage lung cancer detected using lung cancer risk prediction models from recent years, when CT usage has increased. They compared their data with the results of the NLST trial from 2011. In our study, “advanced stage” patients accounted for 72% of randomly detected lung malignancy patients; however, we did not find a significant difference in this ratio compared to the control group, which underwent CT scans considering lung cancer risk factors. In addition, the comparison of the malignancy rates of incidentally detected pulmonary nodules of 8 mm or larger with those detected on CT scans performed in the presence of risk factors in our study is the key factor distinguishing it from the study by Wang et al. [15].

Studies have also found that CT scans performed during the pandemic increased the detection rate of incidental pulmonary nodules, regardless of size. A significant proportion of nodules measuring 6 mm or larger were identified as lesions with a risk of malignancy. This result has been interpreted as suggesting a possible increase in the detection rate of early-stage lung cancer [27]. Our study provides guidance on this point. Although the detection rate of pulmonary nodules has increased, the incidence of malignancy in nodules 8 mm and larger has not changed.

Our study had certain limitations. One of these was that it was not a longitudinal research. The study's strengths lie in its analysis of data from a period when a significant portion of the population underwent CT screening during the pandemic.

## 5. Conclusion

The presence or absence of established risk factors for lung cancer does not influence the incidence of malignancy in pulmonary nodules measuring ≥ 8 mm or greater. Guidelines may not necessitate intricate algorithms; additional research is required to elucidate this matter.

## Figures and Tables

**Table 1:** Distribution and comparison of participants in the two groups according to age and gender.

	**Total**	**Control group** **(n = 158)**	**Study group** **(n = 143)**	**p**
	**Mean ± sd**	**Mean ± sd**	**Mean ± sd**	

Age	65.36 ± 12.31	67.37 ± 11.29	63.14 ± 13.04	**p**=0.009^∗^

	**n (%)**	**n (%)**	**n (%)**	

*Gender*				*p* = 0.422
Female	86 (28.6)	42 (26.6)	44 (30.8)	
Male	215 (71.4)	116 (73.4)	99 (69.2)	

*Note:* Chi-square test was used for categorical variables. Numerical variables did not show normal distribution. Mann–Whitney U test was used. The bold values indicates the statistically significant value.

^∗^ *p* < 0.05.

**Table 2:** Comparison of the two groups according to pulmonary nodule size, presence of malignancy, lung cancer PET stages, and histologic subtypes.

Variables	Control group (*n* = 158)	Study group (*n* = 143)	*x* ^2^/*p*
	*n* (%)	*n* (%)	
*Pulmonary nodule size (mm)*			
8–10	27 (42.9)	**36 (57.1)**	
11–20	63 (56.8)	48 (43.2)	**x** ^2^ **: 8.943**
21–30	**28 (68.3)**	13 (31.7)	**p**=0.037^∗^
> 30	40 (46.5)	46 (53.5)	

*Presence of malignancy*	87 (56.5)	70 (48.9)	*x* ^2^: 2.025 *p* = 0.155

PET stages (n = 157)			
Early stage (Stages I-II)	18 (56.3)	14 (43.8)	
Locally advanced stage (Stage IIIA)	33 (58.9)	23 (41.1)	*x* ^2^: 0.832
Advanced stage (Stages IIIB-IV)	32 (53.3)	28 (46.7)	*p* = 0.842
Metastasis	4 (44.4)	5 (55.6)	

*Histologic subtypes (n = 148)*			
NSCLC	79 (57.2)	59 (42.8)	*x* ^2^: 1126
SCLC	4 (40.0)	6 (60.0)	*p*=0.289

*Note:x* ^2^, chi-square test. Bold values indicate statistically significant values.

Abbreviations: NSCLC, non-small cell lung cancer; SCLC, small cell lung cancer.

^∗^ *p* < 0.05.

**Table 3:** Comparison of pulmonary nodule sizes and malignancy rates according to nodule size between groups.

Pulmonary nodule size		Presence of malignancy (*n* = 154)	*x* ^2^/*p*
		*n* (%)	
8–10 mm		9 (14.3)	
11–20 mm		41 (36.9)	
21–30 mm		**26 (63.4)**	**x** ^2^ **: 99.538**
> 30 mm		**78 (90.7)**	**p**=0.001^∗^

8–10 mm	Control group	5 (18.5)	*x* ^2^: 0.691
	Study group	4 (11.1)	*p*=0.406

11–20 mm	Control group	27 (42.9)	*x* ^2^: 2.192
	Study group	14 (29.2)	*p*=0.139

21–30 mm	Control group	16 (57.1)	*x* ^2^: 1.497
	Study group	10 (76.9)	*p*=0.221

> 30 mm	Control group	39 (97.5)	*x* ^2^: 4.101
	Study group	39 (84.8)	*p*=0.063^†^

*Note:x* ^2^, chi-square test. Statistically significant values are indicated in bold.

^†^Fisher's exact test.

^∗^ *p* < 0.05.

## Data Availability

The data underlying this article will be shared on reasonable request to the corresponding author.
